# Quantitative Proteome Analysis of *Leishmania donovani* under Spermidine Starvation

**DOI:** 10.1371/journal.pone.0154262

**Published:** 2016-04-28

**Authors:** Shalini Singh, Vikash Kumar Dubey

**Affiliations:** Department of Biosciences and Bioengineering, Indian Institute of Technology Guwahati, Assam, India- 781039; Jamia Millia Islamia, INDIA

## Abstract

We have earlier reported antileishmanial activity of hypericin by spermidine starvation. In the current report, we have used label free proteome quantitation approach to identify differentially modulated proteins after hypericin treatment. A total of 141 proteins were found to be differentially regulated with ANOVA P value less than 0.05 in hypericin treated *Leishmania* promastigotes. Differentially modulated proteins have been broadly classified under nine major categories. Increase in ribosomal protein S7 protein suggests the repression of translation. Inhibition of proteins related to ubiquitin proteasome system, RNA binding protein and translation initiation factor also suggests altered translation. We have also observed increased expression of Hsp 90, Hsp 83–1 and stress inducible protein 1. Significant decreased level of cyclophilin was observed. These stress related protein could be cellular response of the parasite towards hypericin induced cellular stress. Also, defective metabolism, biosynthesis and replication of nucleic acids, flagellar movement and signalling of the parasite were observed as indicated by altered expression of proteins involved in these pathways. The data was analyzed rigorously to get further insight into hypericin induced parasitic death.

## Introduction

Leishmaniasis is a vector borne disease caused by a digenetic parasite of genus *Leishmania*. There are few drugs available in the market against leishmaniasis, but they have many limitations, like drug resistance, toxicity, high cost etc. These limitations of available drugs emphasize the need of new and better drug candidates. In search of this, we have identified a novel drug candidate, hypericin, against leishmaniasis [1]. Hypericin was found to be an inhibitor of spermidine synthase of *Leishmania donovani* causing necrotic death of the parasite. Hypericin was found to inhibit the growth of the parasite with IC_50_ value of 18 μM in 24 h. Spermidine was found to play important role in various organisms. In addition to its role in redox metabolism, spermidine was also found to play important role in autophagy and hypusine modification of eukaryotic initiation factor 5A [1]. Analyzing proteome profile of *Leishmania* promastigotes will help us in understanding the global picture of mechanism of hypericin induced cell death. Quantitative proteomics is an important tool for analyzing differential expression of proteins of organisms in different conditions. This further helps in exploring and identifying different mechanisms and new pathways of an organism. Here we have used label free quantification approach for proteome profiling of *Leishmania donovani*. Label free quantification relies on separation of peptides of digested protein through liquid chromatography followed by introduction of ionized peptides into mass spectrometer [2]. Quantitation is based on two methods: one includes measurement in differences of ion intensities like peak area, peak height of the peptide and second is based on the spectral counting of the proteins identified after LC/MS/MS [3]. Label free quantitation is found to be advantageous over labelling base quantitation. It provides exemption from the isotope and fluorescence labelling techniques. Label free quantitation was found to give faster and cleaner results as compared to labelling based quantitation. Label free quantitation is cost effective and shown to have largest dynamic range and highest coverage of proteome as compared to other methods like SILAC, iTRAQ etc [4].

To get a deep insight into the overall mechanism of hypericin induced death of *Leishmania donovani*, it is important to know the global picture of proteome modulation after hypericin treatment. Here we have analyzed proteome of untreated and hypericin treated *Leishmania donovani*.

## Materials and Methods

### Chemicals and cell lines

*Leishmania donovani* (MHOM/IN/2010/BHU1081) strain was a kind gift from Prof. Shyam Sundar, Banaras Hindu University, India. Hypericin and Protease inhibitor cocktail were procured from Sigma Aldrich. Tris-HCl, NaCl etc were of high quality obtained from Himedia. Water, acetonitrile, and formic acid were LC-MS grade and were obtained from Fluka.

### Culture and treatment of *Leishmania* promastigotes

Promastigotes of *Leishmania donovani* were grown in Medium 199 at 25°C. *Leishmania* promastigotes with a cell density of (1X10^6^ cells/ml) were treated with IC_50_ dose of hypericin (18 μM) for 24 h [1]. *Leishmania* promastigotes without any treatment were taken as control.

### Sample preparation and mass spectrometry analysis

Untreated and hypericin treated *Leishmania* promastigotes were analyzed by mass spectrometry. *Leishmania* promastigotes (1X10^6^ cells/ml) were harvested and dissolved in lysis buffer (50 mM tris-Cl, 150 mM NaCl, and protease inhibitor cocktail). Promastigotes were lysed using ultrasonication with pulse cycle of 2 sec ON and 10 sec OFF for 5 min in lysis buffer. After lysis, promastigotes were centrifuged at 12,000 rpm for 20 min to remove the cell debris. Supernatant was collected and again centrifuged at 12,000 rpm for 20 min. Protein was precipitated from lysate of *Leishmania* promastigotes using acetone precipitation. Further, protein concentration was measured using bicinchoninic acid (BCA) analysis. Alkylation and reduction of sample was performed and equal amount (30 μg) of each sample was subjected to digestion by trypsin. After this, samples were dissolved in 15 μl of 2% acetonitrile and 0.1% formic acid. Each sample (1 μl i.e. 300 ng) is subjected to reverse phase liquid chromatography on 1200, 1D nano-LC (Agilent) system for 180 min which is then followed by acquisition of data on LTQ-Orbitrap-MS (LTQ–Orbitrap Discovery, Thermo). C18 analytical column was used for the experiment. Two solvents, solvent A (100% water with 0.1% formic Acid) and solvent B (80% acetonitrile and 20% water with 0.1% formic acid) were used where gradient was varied from 11% B to 100% from 0 to 160 min and then again percentage of solvent B was kept as 11% from 160 min to 180 min [5, 6]. The experiment was done in triplicate.

### Analysis of mass spectrometry data and label free quantitation

The data obtained from mass spectrometry was analyzed by using Progenesis QI for proteomics [7, 8]. The Progenesis QI is the new name for Progenesis LC-MS and is available from Nonlinear Dynamics, United Kingdom. The software identifies the peak and creates peak models retaining important information of quantification and position. Further, the reproducibility of the experiment was increased by aligning the results of different runs by identifying a best reference run. Aligned runs were analyzed to create a data set containing the information about all the peaks identified in all sample runs. After quantification of ion abundance from each run, the data was normalized to compare the results from different runs. Statistical analysis was done by using ANOVA. Peptide ions were further identified by using MASCOT MS/MS ion search tool. The *Leishmania* database used for MASCOT search contains 17,599 sequences. Peptide mass tolerance and fragment mass tolerance was kept at ±1.2 Da and ±0.6 Da respectively. Unique peptides were considered for protein quantification and score was analyzed which reflects the confidence of the identified proteins during the database search [9, 10]. Proteins were classified into major functional group based on their Uniprot accession number.

### Protein-protein interaction and phylogenetic relationship analysis

Differentially upregulated proteins above 1.5 fold and down regulated protein below 0.9 fold were taken to analyze the interaction between them. Protein-protein interaction was analyzed by using STRING database. STRING (Search Tool for the Retrieval of Interacting Genes) is a database containing known and predicted associations between proteins. Protein interactions in STRING are not only based on direct and physical association of proteins but also on their genetic interactions and their involvement in subsequent catalysis steps in metabolic processes [11, 12]. STRING has also provided the coexpression data of several proteins involved in network interaction. The pylogenetic relationship between proteins showing protein protein interaction with and their functional partners were analyzed by first aligning the sequences in Clustal Omega and then subjecting the aligned sequences to generate the phylogenetic relationship by using Clustal W2- Phylogeny [13, 14].

## Results

### Distribution of proteins being altered after hypericin treatment into major classes

**Fig 1:**
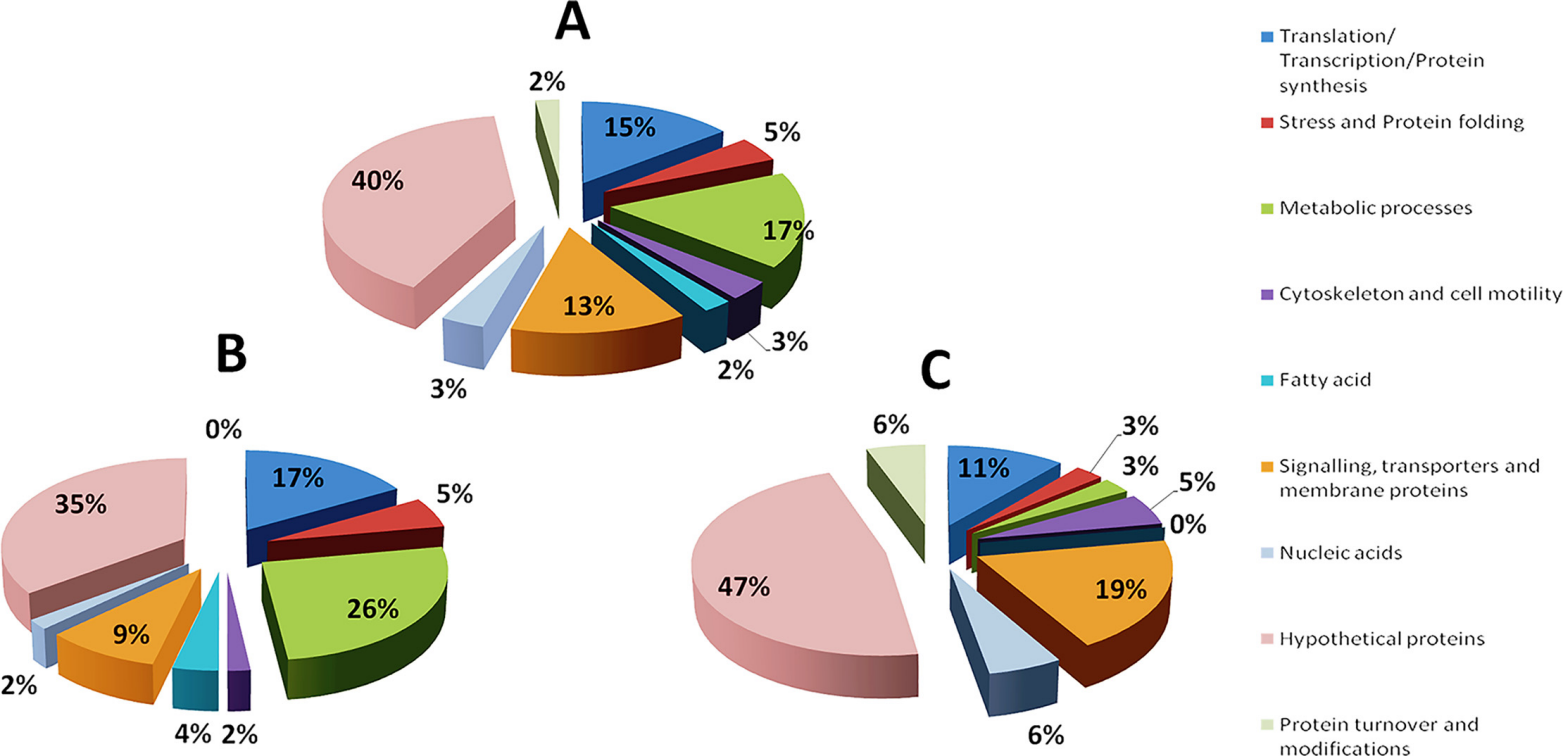
Pie chart showing relative distribution of differentially modulated proteins (up regulated above 1.5 fold and down regulated below 0.9 fold with ANOVA value less than 0.05) of *Leishmania donovani* after hypericin treatment. **(A)** Pie chart of total proteins being altered after hypericin treatment showing their relative distribution among 9 major categories. **(B)** Pie chart of up regulated proteins after hypericin treatment showing their relative distribution. **(C)** Pie chart of down regulated proteins after hypericin treatment showing their relative distribution.

**Fig 2:**
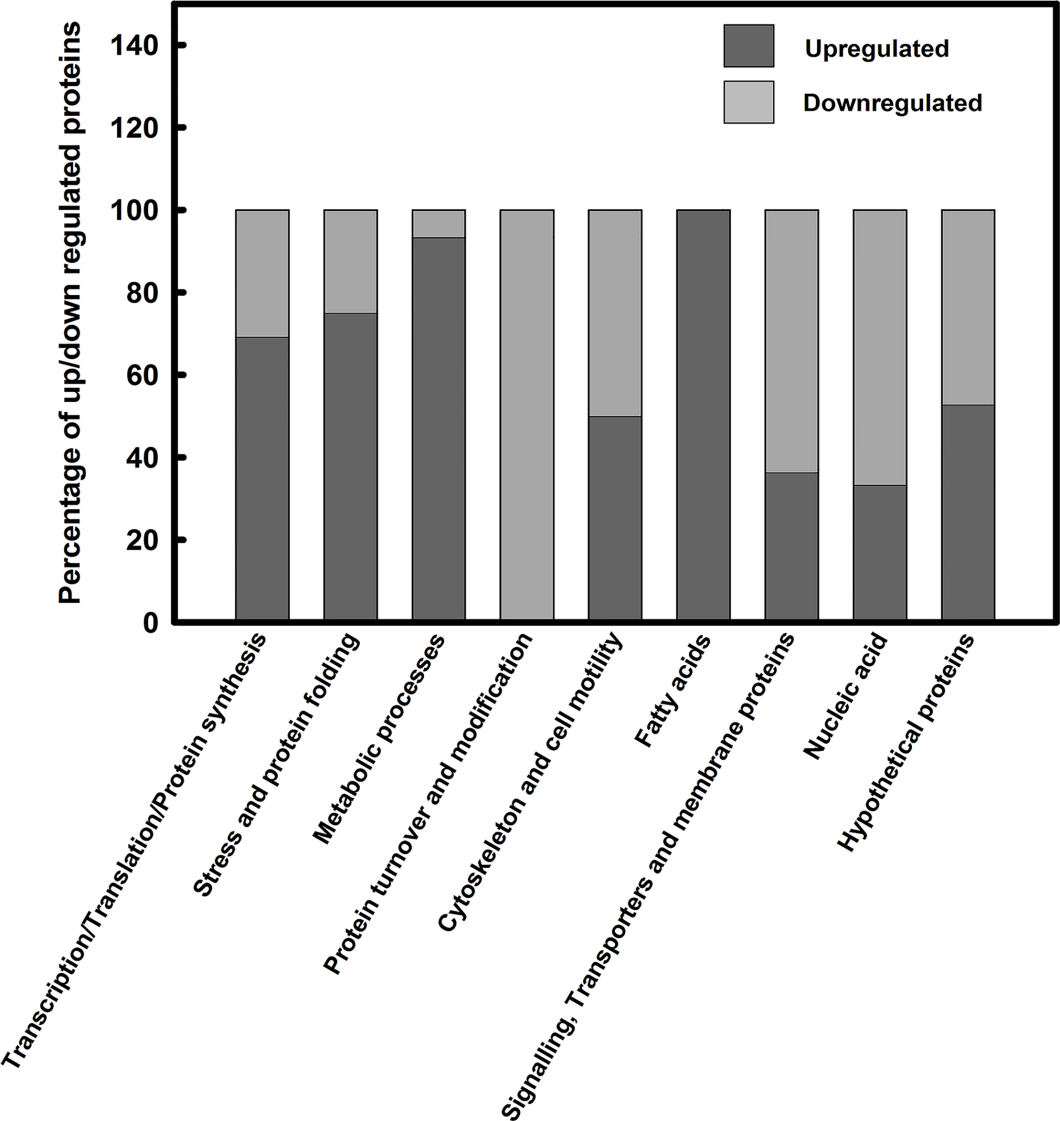
Bar diagram showing percentage of up-regulated and down-regulated (up-regulated above 1.5 fold and down-regulated below 0.9 fold with ANOVA value less than 0.05) proteins of *Leishmania donovani* after hypericin treatment based on their functional classification.

Differentially modulated proteins are categorised into nine major classes. These classes are: Protein synthesis, stress and protein folding, metabolic processes, protein turnover and modification, cytoskeleton and cell motility, fatty acids, signalling, transporters and membrane proteins, nucleic acid and hypothetical proteins. Relative distribution among different classes of total proteins being altered after hypericin treatment is shown by plotting a pie chart (Fig 1A). A total of 141 proteins were identified with ANOVA value less than 0.05 and out of this 109 were up-regulated 32 were down-regulated. However, for analysis we have considered those proteins (90 proteins) which are differentially expressed with up-regulation above 1.5 fold and down-regulation below 0.9 fold. Out of total altered protein, 15% are in the category of protein synthesis, 5% in stress and protein folding, 17% under metabolic processes, 2% in protein turnover and modification, 3% in cytoskeleton and cell motility, 2% related to fatty acids, 13% related to signalling, transporters and membrane proteins, 3% in nucleic acids and 40% hypothetical proteins (Fig 1A). The relative distribution of up regulated proteins (up regulated above 1.5 fold with ANOVA value less than 0.05) is also checked by plotting pie chart (Fig 1B). The percentage of up regulated proteins out of total up regulated proteins are: 17% protein synthesis, 5% stress and protein folding, 26% metabolic processes, 0% protein turnover and modification, 2% cytoskeleton and motility, 4% fatty acids, 9% signalling, transporters and membrane proteins, 2% nucleic acids and 35% hypothetical proteins (Fig 1B). Out of total proteins being down regulated (down regulated below 0.9 fold with ANOVA value less than 0.05), 11% belong to the category of protein synthesis, 3% stress and folding, 3% metabolic processes, 6% protein turnover and modification, 5% cytoskeleton and cell motility, 0% fatty acids, 19% signalling, transporters and membrane proteins, 6% nucleic acids and 47% hypothetical proteins (Fig 1C). The up-regulated and down-regulated proteins have been combined in each functional class and subsequently their percentage has been calculated. The plot of percentage of up and down-regulated proteins in each class is shown in Fig 2. This is important to analyse the percentage of protein being up-regulated and down-regulated in each functionally classified group.

### Differential regulation of proteins related to protein synthesis

A 2.96 fold increase in elongation factor 1 alpha was observed. There was slight increase (1.68 fold) in eukaryotic translation initiation factor. Ribosomal protein S7, ribosomal protein L15 and histone H2B has shown 5.26, 1.99 and 5.06 fold respective increase in expression after hypericin treatment. In *E*. *coli* ribosomal protein S7 is involved in the formation of 30S subunit of ribosome. It is also found to be translation repressor and it’s over expression retarded growth in *E*. *coli* [15]. There is an increased expression of poly (A)-binding protein and threonyl-tRNA synthetase after hypericin treatment (Table 1). Proteins which show decreased expression after hypericin treatment are 60S ribosomal protein L12 (0.83 fold), splicing factor ptsr1-like protein (0.73 fold), RNA-binding protein (0.63 fold) and translation initiation factor (0.75 fold) (Table 2).

**Table 1:** Distribution of up regulated proteins after hypericin treatment into major categories. Any increase above fold 1 indicates up-regulation. The table includes up-regulated proteins with ANOVA value less than 0.05.

Accession	Anova (P*)	Folds	Peptides(Peptides in quantitation)	Score	Description	Average Normalized Abundance	
						Treated	Control
**Transcription/Translation/Protein synthesis**							
15788964	1.08e-003	2.96	29 (6)	702.34	elongation factor 1-alpha, partial	5.42e+007	1.83e+007
322496059	6.23e-003	5.26	10 (1)	230.03	ribosomal protein S7, putative	3.47e+006	6.59e+005
322502154	0.01	1.45	16 (4)	395.57	40S ribosomal protein S3, putative	9.68e+006	6.68e+006
322497920	0.01	1.38	14 (2)	177.76	eukaryotic translation initiation factor 1A, putative	2.08e+006	1.51e+006
322501313	0.02	1.99	11 (4)	151.09	ribosomal protein L15, putative	4.21e+006	2.12e+006
322498799	0.04	1.26	13 (3)	387.10	la RNA binding protein, putative	3.17e+006	2.52e+006
322498921	8.95e-003	1.48	23 (6)	322.74	RNA helicase, putative	7.19e+006	4.88e+006
322499565	1.17e-003	1.64	18 (4)	309.36	poly(A)-binding protein, putative	3.10e+006	1.89e+006
322502963	0.05	1.52	17 (7)	345.52	threonyl-tRNA synthetase, putative	5.28e+006	3.47e+006
322500575	0.04	1.68	10 (4)	295.75	eukaryotic translation initiation factor, putative	4.08e+006	2.43e+006
322497525	0.03	1.39	26 (8)	571.80	leucyl-tRNA synthetase, putative	6.28e+006	4.53e+006
322497888	0.04	1.34	16 (3)	133.77	glutaminyl-tRNA synthetase, putative	6.58e+006	4.93e+006
322502866	8.01e-004	1.31	15 (5)	318.63	ATP-dependent DEAD-box RNA helicase, putative	1.29e+007	9.82e+006
322496663	0.04	1.56	13 (2)	201.10	ATP-dependent DEAD/H RNA helicase, putative	4.39e+006	2.82e+06
68235787	3.18e-004	5.06	9 (2)	197.04	histone H2B protein	1.07e+07	2.11e+06
322498355	0.04	1.54	7 (1)	197.38	prolyl-tRNA synthetase, putative, partial	7.46e+05	4.83e+05
322503349	0.03	1.25	19 (4)	212.95	polyadenylate-binding protein 1, putative	3.00e+007	2.39e+007
322503415	8.95e-003	1.45	22 (2)	234.71	Mitochondrial elongation factor G, putative	4.33e+006	2.99e+006
322503916	0.03	1.32	8 (2)	105.09	branch point binding protein, putative	2.37e+006	1.79e+006
**Stress and protein folding**							
322502093	0.02	3.27	54 (3)	1817.23	heat shock protein 83–1	1.74e+007	5.32e+006
12232034	0.04	1.24	32 (1)	319.92	chaperonin TCP20	2.16e+006	1.73e+006
123669	0.03	3.72	33 (2)	908.40	RecName: Full = Heat shock protein 83; Short = HSP 83; AltName: Full = HSP 90	3.88e+006	1.04e+006
322497565	0.03	1.10	26 (5)	405.24	chaperonin TCP20, putative	1.44e+007	1.32e+007
322503363	0.04	2.43	11 (2)	289.24	stress-inducible protein STI1 homolog	4.15e+006	1.71e+006
322504075	0.03	1.28	24 (8)	729.49	protein disulfide isomerase	4.07e+007	3.17e+007
145411494	0.02	1.07	11 (2)	215.58	cytoplasmic tryparedoxin peroxidase	1.08e+007	1.00e+007
**Metabolic processes**							
16797868	0.03	1.48	39 (9)	1033.69	glucose-regulated protein 78, partial	2.76e+007	1.87e+007
322502940	0.03	1.51	53 (18)	961.31	NADH-dependent fumarate reductase, putative	3.00e+007	1.99e+007
322502306	0.04	1.44	28 (2)	645.87	succinyl-coA:3-ketoacid-coenzyme A transferase, mitochondrial precursor, putative	2.13e+006	1.47e+006
322500910	0.04	1.15	24 (9)	562.03	ATP-dependent phosphofructokinase	1.15e+007	9.96e+006
322500647	1.13e-003	1.41	4 (2)	90.64	glucose 6-phosphate N-acetyltransferase, putative	4.98e+006	3.52e+006
322503662	0.02	1.23	17 (3)	552.53	succinyl-CoA ligase [GDP-forming] beta-chain, putative	5.32e+006	4.34e+006
322503492	2.88e-03	1.74	19 (2)	186.23	N-acetyltransferase subunit Nat1, putative	5.97e+06	3.43e+06
148913117	0.04	1.62	15 (3)	181.43	glycogen synthase kinase 3 short	4.71e+06	2.91e+06
322497288	4.36e-003	1.27	37 (9)	657.91	pyruvate phosphate dikinase, putative	1.02e+007	8.04e+006
322502415	9.44e-004	1.60	13 (1)	331.12	malate dehydrogenase	5.72e+006	3.57e+006
322497392	1.55e-003	1.96	9 (3)	158.65	cytochrome c oxidase subunit IV	9.15e+006	4.66e+006
322502326	0.02	1.35	14 (6)	300.21	isocitrate dehydrogenase, putative	7.32e+006	5.40e+006
322498469	6.07e-003	1.21	14 (4)	232.64	glycosomal malate dehydrogenase	4.17e+006	3.45e+006
190335775	0.04	1.33	33 (8)	1021.25	enolase	1.05e+007	7.86e+006
322502960	5.32e-003	1.71	17 (4)	394.15	mitochondrial processing peptidase, beta subunit, putative	1.06e+007	6.18e+006
322503715	0.02	1.67	22 (12)	382.32	2-oxoglutarate dehydrogenase E1 component, putative	6.41e+006	3.85e+006
322497501	0.04	1.26	19 (5)	311.09	mitochondrial processing peptidase alpha subunit, putative	6.13e+007	4.85e+007
322500587	9.51e-003	1.55	9 (3)	127.36	2-oxoglutarate dehydrogenase, E2 component, dihydrolipoamide succinyltransferase, putative	3.64e+006	2.35e+006
322496602	0.02	2.14	5 (1)	60.63	dihydrofolate reductase-thymidylate synthase	7.01e+005	3.28e+005
322500467	0.02	1.69	7 (2)	119.16	phenylalanine-4-hydroxylase, putative	2.50e+006	1.48e+006
322498779	0.02	1.78	8 (2)	109.23	mitochondrial processing peptidase alpha subunit, putative	1.87e+006	1.05e+006
322502201	0.03	1.56	6 (2)	105.24	cysteine conjugate beta-lyase, aminotransferase-like protein (pyruvate metabolism)	7.29e+006	4.69e+006
322501324	0.02	1.52	22 (5)	491.49	5-methyltetrahydropteroyltriglutamate-homocysteine S-methyltransferase, putative (Methionine metabolism)	8.51e+006	5.61e+006
322502231	4.73e-003	1.16	6 (2)	173.98	peptidase M20/M25/M40, putative	3.98e+006	3.42e+006
322499163	0.04	1.66	14 (2)	199.28	(H+)-ATPase G subunit, putative	2.23e+006	1.35e+006
**Protein turnover and modification**							
322502952	0.03	1.27	9 (2)	426.04	ubiquitin-conjugating enzyme e2, putative	1.14e+007	8.93e+006
322503495	7.69e-003	1.22	40 (15)	898.84	Transitional endoplasmic reticulum ATPase, putative	3.49e+007	2.87e+007
322500536	0.03	1.30	9 (6)	115.89	X-pro, dipeptidyl-peptidase,serine peptidase, Clan SC, family S15, putative	4.21e+006	3.25e+006
478212838	0.01	1.26	10 (5)	84.48	cysteine protease b	1.01e+007	8.01e+006
322497214	0.03	1.19	12 (6)	198.50	proteasome alpha 7 subunit, putative	9.22e+007	7.77e+007
322497557	0.04	1.46	10 (4)	198.12	ubiquitin-conjugating enzyme-like protein	6.97e+06	4.76e+06
**Cytoskeleton and cell motility**							
322497027	5.37e-003	1.40	23 (6)	295.01	paraflagellar rod component, putative	2.84e+006	2.03e+006
299829504	0.01	2.16	42 (10)	1952.49	beta tubulin	1.57e+008	7.27e+007
6708172	0.03	1.43	8 (4)	300.18	ADP-ribosylation factor-like protein 3A	2.60e+006	1.82e+006
322496512	5.60e-03	1.62	16 (4)	184.04	prefoldin subunit, putative	1.03e+07	6.38e+06
**Fatty acids**							
322499210	0.05	1.47	28 (9)	710.85	3-ketoacyl-CoA thiolase-like protein	1.39e+007	9.48e+006
322497652	1.85e-003	14.86	4 (1)	109.81	fatty acid elongase, putative	8.63e+005	5.80e+004
322499741	0.02	1.47	7 (2)	104.12	3-oxo-5-alpha-steroid 4-dehydrogenase, putative	4.25e+006	2.89e+006
322499090	2.68e-003	2.07	6 (2)	93.29	farnesyl pyrophosphate synthase	2.07e+006	9.96e+005
**Signalling, Transporters and membrane proteins**							
151413555	7.83e-003	1.46	21 (5)	392.70	adenylate nucleotide translocase	1.33e+007	9.11e+006
5813867	0.04	1.62	12 (1)	300.51	putative pteridine transporter FT3	1.48e+007	9.10e+006
322503191	0.01	1.20	15 (5)	406.76	Gim5A protein, putative	1.54e+007	1.28e+007
322499307	0.01	1.58	8 (1)	112.93	vacuolar proton translocating ATPase subunit A, putative	2.86e+006	1.80e+006
322497687	0.04	1.64	6 (1)	62.86	ADP/ATP mitochondrial carrier-like protein	4.80e+005	2.92e+005
322497170	0.01	1.25	5 (3)	78.82	rab1 small GTP-binding protein, putative	2.59e+006	2.07e+006
322502740	0.03	1.47	23 (4)	312.96	vacuolar ATP synthase catalytic subunit A, putative	2.01e+006	1.37e+006
322501447	0.04	1.30	10 (2)	237.88	vacuolar-type proton translocating pyrophosphatase 1, putative	1.14e+007	8.76e+006
322496444	5.23e-003	1.57	17 (4)	466.13	ATPase alpha subunit	1.63e+007	1.04e+007
322498413	0.02	1.26	11 (4)	189.73	protein kinase, putative	2.55e+06	2.02e+06
322498952	9.58e-003	1.12	17 (5)	326.04	centromere/microtubule binding protein cbf5, putative	3.45e+006	3.09e+006
322498624	0.02	1.24	15 (4)	201.35	cytidine triphosphate synthase, putative	4.05e+006	3.26e+006
322497534	0.03	1.79	17 (3)	272.86	adenylosuccinate synthetase, putative (purine metabolism)	1.30e+007	7.29e+006
**Hypothetical proteins**							
322502830	0.05	1.41	5 (3)	60.28	unnamed protein product, partial	2.58e+006	1.82e+006
322497208	0.04	1.53	5 (2)	58.37	hypothetical protein, conserved	2.25e+006	1.47e+006
322501229	0.04	1.31	4 (2)	54.47	hypothetical protein, conserved	2.78e+006	2.12e+006
322501847	0.02	2.62	6 (2)	27.39	hypothetical protein, unknown function	2.71e+006	1.03e+006
322498448	0.01	4.65	4 (1)	26.88	hypothetical protein, conserved	4.00e+005	8.60e+004
322502714	0.04	1.33	6 (1)	75.50	hypothetical protein, conserved	1.68e+006	1.27e+006
322497611	1.45e-003	1.57	3 (1)	77.89	hypothetical protein, conserved	4.82e+006	3.07e+006
322497181	0.02	1.62	6 (1)	73.13	hypothetical protein, conserved	7.13e+006	4.39e+006
322499167	0.01	1.53	4 (1)	72.42	hypothetical protein, conserved	2.47e+006	1.61e+006
322498140	4.14e-003	1.27	5 (2)	85.40	hypothetical protein, conserved	4.58e+006	3.62e+006
322502559	8.21e-003	1.29	8 (5)	96.53	hypothetical protein, conserved	1.62e+007	1.26e+007
322500809	4.22e-004	2.53	8 (1)	106.38	hypothetical protein, conserved	1.33e+006	5.25e+005
322502778	0.04	1.63	5 (2)	113.50	hypothetical protein, conserved	5.08e+006	3.11e+006
322503307	3.70e-005	2.31	10 (4)	117.83	hypothetical protein, conserved	1.12e+007	4.84e+006
322500012	0.01	1.57	5 (2)	123.32	hypothetical protein, conserved	2.54e+006	1.62e+006
322503312	2.62e-003	2.78	12 (1)	126.28	hypothetical protein, conserved	1.64e+006	5.92e+005
322498829	0.03	1.31	15 (2)	130.64	hypothetical protein, conserved	4.36e+007	3.32e+007
322498659	0.03	1.41	10 (4)	129.48	hypothetical protein, conserved	6.22e+006	4.42e+006
322501903	0.03	1.89	14 (1)	151.47	hypothetical protein, conserved	4.06e+006	2.14e+006
322502401	0.01	1.96	16 (4)	163.46	hypothetical protein, conserved	2.57e+006	1.31e+006
322496063	0.04	1.33	21 (8)	212.80	hypothetical protein, conserved	9.06e+006	6.82e+006
322497523	0.03	1.20	25 (7)	207.01	hypothetical protein, unknown function	2.06e+007	1.71e+007
322502955	0.02	1.75	11 (4)	229.44	hypothetical protein, conserved	2.20e+007	1.26e+007
322496696	0.05	1.16	20 (5)	228.43	hypothetical protein, conserved	5.87e+006	5.05e+006
322499284	0.02	2.30	8 (1)	254.93	hypothetical protein, conserved	7.81e+005	3.40e+005
322503433	0.01	4.20	16 (5)	240.22	hypothetical protein, conserved	1.25e+007	2.97e+006
322501739	0.01	1.30	11 (4)	374.99	hypothetical protein, conserved	1.26e+007	9.70e+006
322496809	0.03	1.58	49 (12)	379.88	hypothetical protein, conserved	2.48e+007	1.57e+007
322496585	1.58e-003	1.75	38 (6)	312.42	hypothetical protein, conserved	1.00e+007	5.73e+006
322496901	0.04	1.35	28 (4)	395.31	hypothetical protein, conserved	4.16e+007	3.07e+007
322496996	0.04	1.44	20 (6)	555.79	hypothetical protein, conserved	1.72e+007	1.20e+007
322500235	0.04	1.21	27 (5)	193.20	hypothetical protein, conserved	7.68e+006	6.36e+006
322502308	0.02	1.51	13 (1)	186.52	hypothetical protein, conserved	2.30e+06	1.52e+06

**Table 2:** Distribution of down-egulated proteins after hypericin treatment into major categories. Any decrease below fold 1 indicates down-regulation. The table includes down-regulated proteins with ANOVA value less than 0.05.

Accession	Anova (P*)	Folds	Peptides (Peptides in quantitation)	Score	Description	Average Normalized Abundance	
						Treated	Control
**Transcription/Translation/Protein synthesis**				
322500439	0.05	0.95	12 (3)	337.51	ribosomal protein S20, putative	3.23e+006	3.39e+006
322498212	0.05	0.75	13 (6)	160.66	translation initiation factor, putative	3.54e+006	4.72e+006
322500281	0.04	0.63	8 (1)	104.82	RNA-binding protein, putative	4.72e+005	7.47e+005
322503038	0.01	0.83	7 (4)	78.94	60S ribosomal protein L12, putative	7.11e+006	8.54e+006
322496746	2.71e-003	0.73	23 (6)	366.99	splicing factor ptsr1-like protein	6.50e+006	8.89e+006
**Stress and protein folding**							
322501328	0.02	0.46	2 (1)	33.66	cyclophilin, putative	1.57e+006	3.39e+006
**Metabolic processes**							
322500964	4.77e-003	0.73	10 (3)	155.73	2-hydroxy-3-oxopropionate reductase, putative	7.86e+006	1.08e+007
**Protein turnover and modification**							
322500105	0.01	0.76	7 (3)	283.01	proteasome alpha 7 subunit, putative	3.38e+006	4.44e+006
322503519	0.02	0.87	9 (3)	415.66	proteasome alpha 1 subunit, putative	6.10e+006	7.01e+006
**Cytoskeleton and cell motility**							
322497455	4.71e-003	0.70	11 (3)	105.39	flagellar radial spoke protein, putative	1.93e+006	2.76e+006
322498424	0.04	0.50	19 (6)	224.79	C-terminal motor kinesin, putative	1.43e+007	2.86e+007
**Signalling, Transporters and membrane proteins**							
322498892	0.02	0.55	10 (2)	95.98	vesicule-associated membrane protein, putative	1.42e+006	2.57e+006
325973870	9.88e-003	0.65	14 (1)	453.56	leishmanolysin, partial	9.56e+005	1.47e+006
322501606	0.02	0.79	5 (2)	115.91	ADP-ribosylation factor, putative	6.56e+006	8.30e+006
322501308	0.02	0.90	5 (3)	262.66	ATP synthase, epsilon chain, putative	1.00e+007	1.11e+007
322500321	0.04	0.76	8 (3)	71.71	protein kinase, putative	3.66e+006	4.83e+006
322501646	2.03e-004	0.70	4 (2)	55.93	protein kinase, putative	6.65e+007	9.50e+007
3724134	2.84e-003	0.87	5 (1)	178.77	HASPB1 protein	9.59e+006	1.10e+007
322497143	0.03	0.67	8 (3)	193.43	small GTP-binding protein Rab11, putative	4.62e+06	6.81e+06
**Nucleic acid**							
322503524	0.04	0.72	8 (5)	155.97	universal minicircle sequence binding protein (UMSBP), putative	1.80e+007	2.51e+007
322497959	0.03	0.87	9 (3)	125.16	aspartate carbamoyltransferase, putative	2.86e+006	3.28e+006
**Hypothetical proteins**							
322503652	7.08e-003	0.67	3 (1)	60.32	hypothetical protein, conserved	9.11e+005	1.36e+006
322499442	0.04	0.84	11 (4)	74.57	hypothetical protein, conserved	1.80e+006	2.14e+006
322502261	0.01	0.64	8 (3)	72.20	hypothetical protein, conserved	2.12e+006	3.32e+006
322501879	5.33e-003	0.82	6 (2)	91.16	hypothetical protein, conserved	4.66e+007	5.69e+007
322496451	1.66e-004	0.66	6 (3)	90.96	hypothetical protein, conserved	1.98e+008	2.98e+008
322502124	0.01	0.88	5 (4)	188.65	hypothetical protein, conserved	1.96e+07	2.21e+07
322502229	0.03	0.36	9 (1)	187.25	hypothetical protein, conserved	2.88e+05	7.96e+05
322501950	0.04	0.71	11 (3)	96.42	hypothetical protein, conserved	2.55e+006	3.60e+006
322496438	0.01	0.74	4 (1)	110.62	hypothetical protein, conserved	1.44e+007	1.95e+007
322498397	0.01	0.73	6 (2)	109.81	hypothetical protein, conserved	8.78e+006	1.20e+007
322503852	5.53e-003	0.83	11 (7)	113.24	hypothetical protein, conserved	8.56e+006	1.03e+007
322498975	1.17e-003	0.81	7 (3)	123.07	hypothetical protein, conserved	1.17e+006	1.45e+006
322501425	0.05	0.57	13 (4)	159.34	hypothetical protein, conserved	1.48e+006	2.61e+006
322498169	1.02e-003	0.61	12 (4)	172.14	hypothetical protein, conserved	5.79e+006	9.47e+006
322499746	0.03	0.47	15 (2)	214.38	hypothetical protein, conserved	5.84e+005	1.23e+006
322503960	0.04	0.79	18 (4)	226.67	hypothetical protein, conserved	1.61e+007	2.03e+007
322502578	0.03	0.91	31 (12)	256.67	hypothetical protein, conserved	5.50e+007	6.05e+007
322501429	9.67e-003	0.74	18 (7)	353.79	hypothetical protein, unknown function	1.21e+007	1.64e+007

### Altered modulation of proteins related to stress and protein folding

Increase in expression levels of some heat shock proteins (Hsp) was observed. Heat shock proteins are also called stress proteins. Heat shock proteins play various roles in cells, for example, they are involved in providing protection to cells against stress, signalling and cell cycle control etc [16]. There was 3.27 fold increase in the expression of Hsp 83–1 and 3.72 fold increase in Hsp90 after hypericin treatment. Other stress related protein such as stress-inducible protein STI1 homolog was also found to show increased expression (2.43 fold) after hypericin treatment (Table 1). However, cyclophilin (0.46 fold) expression was significantly decreased after hypericin treatment (Table 2).

### Alteration in expression of proteins involved in metabolic processes

Expression of several proteins involved in carbohydrate metabolism, amino acid metabolism and pyruvate metabolism was found to be altered due to hypericin. Several enzymes involved in glucose metabolism were shown to have increase expression after hypericin treatment. Enzymes, like NADH-dependent fumarate reductase (1.51 fold), malate dehydrogenase (1.60 fold), cytochrome c oxidase subunit IV (1.96 fold), 2-oxoglutarate dehydrogenase E1 component (1.67 fold) and 2-oxoglutarate dehydrogenase, E2 component, dihydrolipoamide succinyltransferase (1.55 fold) show altered expression (Table 1). However 2-hydroxy-3-oxopropionate reductase was found to show decreased expression in hypericin treated *Leishmania* promastigotes. Several enzymes involved in mitochondrial biogenesis were also found to be increased. Mitochondrial processing peptidase, beta subunit was increased by 1.71 fold and mitochondrial processing peptidase alpha subunit was increased by 1.78 fold in promastigotes treated with IC_50_ value of hypericin for 24 h. Certain enzymes of amino acid biosynthesis and metabolism were also found to be up regulated. Phenylalanine-4-hydroxylase (1.69 fold) and 5-methyltetrahydropteroyltriglutamate-homocysteine S-methyltransferase (1.52 fold) were shown increased expression after hypericin treatment. Decreased expression of 2-hydroxy-3-oxopropionate reductase (0.73 fold) was found to be decreased in hypericin treated *Leishmania* promastigotes (Table 2).

### Change in expression of enzymes involved in protein turnover, processing and modification was observed after hypericin treatment

The enzymes involved in protein turnover and modification were found to be altered in promastigotes treated with hypericin for 24 h. Proteasome alpha 7 has shown 0.76 fold decrease expression after hypericin treatment. Also, proteasome alpha 1 subunit expression was decreased to some extent (0.87 fold) (Table 2). Proteasome alpha 7 subunit and proteasome alpha 1 subunit are components of ubiquitin proteasome system. Inhibitors of ubiquitin proteasome system have led to the accumulation of unhypusinated eIF5A [17].

### Differential expression of proteins involved in cytoskeleton and cell motility

Some of the proteins involved in cytoskeleton formation and cell motility were altered. Expression of beta tubulin was increased 2.16 fold in promastigotes treated with hypericin for 24 h (Table 1). However, proteins involved in cellular motility were found to show decreased expression after hypericin treatment. C-terminal motor kinesin and flagellar radial spoke protein has shown significant decrease of 0.50 fold and 0.70 fold respectively (Table 2). C-terminal motor kinesin is known to be involved in ATP hydrolysis, spindle assembly during cell division, flagellar movement etc [18]. Radial spoke is an essential component of motile cilia and flagella. Proteins present in the radial spokes are also known contain domains associated with signal transduction like Ca^2+^ domain, A-protein kinase domain, nucleotide binding domain etc [19].

### Change in expression of proteins involved in fatty acid biosynthesis

Proteins involved in fatty acid synthesis shows increased expression after hypericin treatment. Fatty acid elongase expression was increased 14.86 fold in promastigotes treated with hypericin for 24 h. Also, the expression of farnesyl pyrophosphate was found to be higher (2.07 fold) after hypericin treatment (Table 1).

### Alteration in expression of proteins involved in signalling, transport and membrane proteins

There was alteration in expression of signalling proteins, transporters, and membrane proteins in promastigotes treated with IC_50_ dose of hypericin for 24 h. Pteridine transporter FT3 (1.62 fold), vacuolar proton translocating ATPase subunit A (1.58 fold), ADP/ATP mitochondrial carrier-like protein (1.64 fold), ATPase alpha subunit (1.57 fold) were found to be up regulated after hypericin treatment of promastigotes for 24 h (Table 1). Many signalling proteins and transporters show decreased expression after hypericin treatment. The downregulation of vesicule-associated membrane protein (0.55 fold), leishmanolysin (0.65 fold), ADP-ribosylation factor (0.79 fold), ATP synthase, epsilon chain (0.90 fold), protein kinase (Accession number: 322500321) (0.76 fold), protein kinase (Accession number: 322501646) (0.70 fold) and HASPB1 protein (0.87 fold) was observed in promastigotes treated with hypericin for 24 h (Table 2).

### Altered expression of proteins involved in nucleic acid synthesis and metabolism

Altered expression of proteins involved in nucleic acid biosynthesis and metabolism was observed. Adenylosuccinate synthetase and dihydrofolate reductase-thymidylate synthase were up regulated by 1.79 fold and 2.14 fold after hypericin treatment (Table 1). Some of the proteins were also down regulated such as universal minicircle sequence binding protein (UMSBP) (0.72 fold) and aspartate carbamoyltransferase (0.87 fold) (Table 2).

### Protein-protein interaction of differentially modulated proteins and phylogenetic analysis

**Fig 3:**
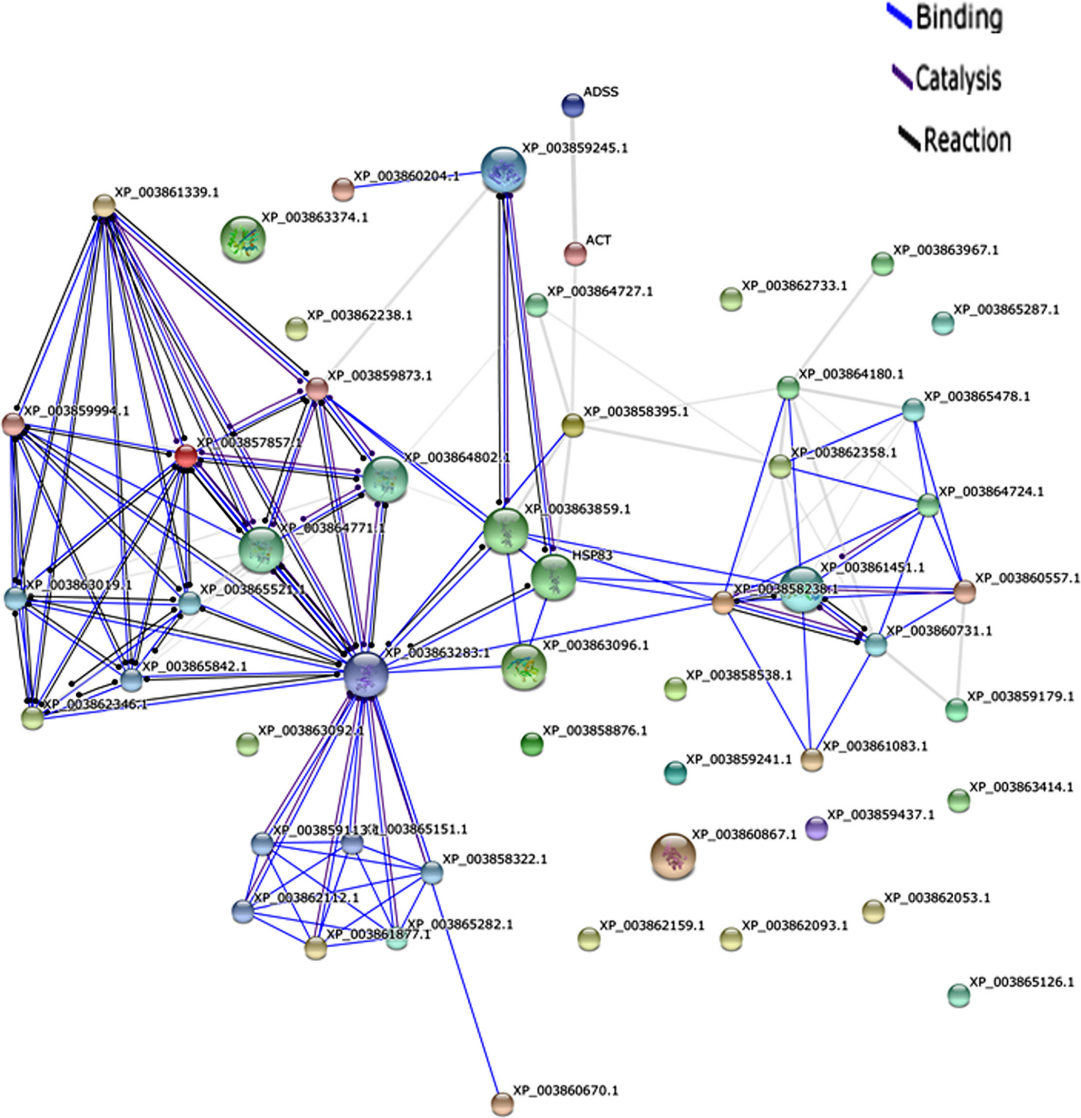
Protein-protein interaction diagram of annotated proteins up regulated above 1.5 fold and down regulated below 0.9 fold with ANOVA value less than 0.05. Protein-protein interactions between the proteins were analyzed by using STRING database. Here the thickness of the lines represents the strength of the association between two proteins. The mode of interaction of each protein is represented by different colours. Also, the directionality of action is represented by the presence of dots at the end of connecting lines.

**Fig 4:**
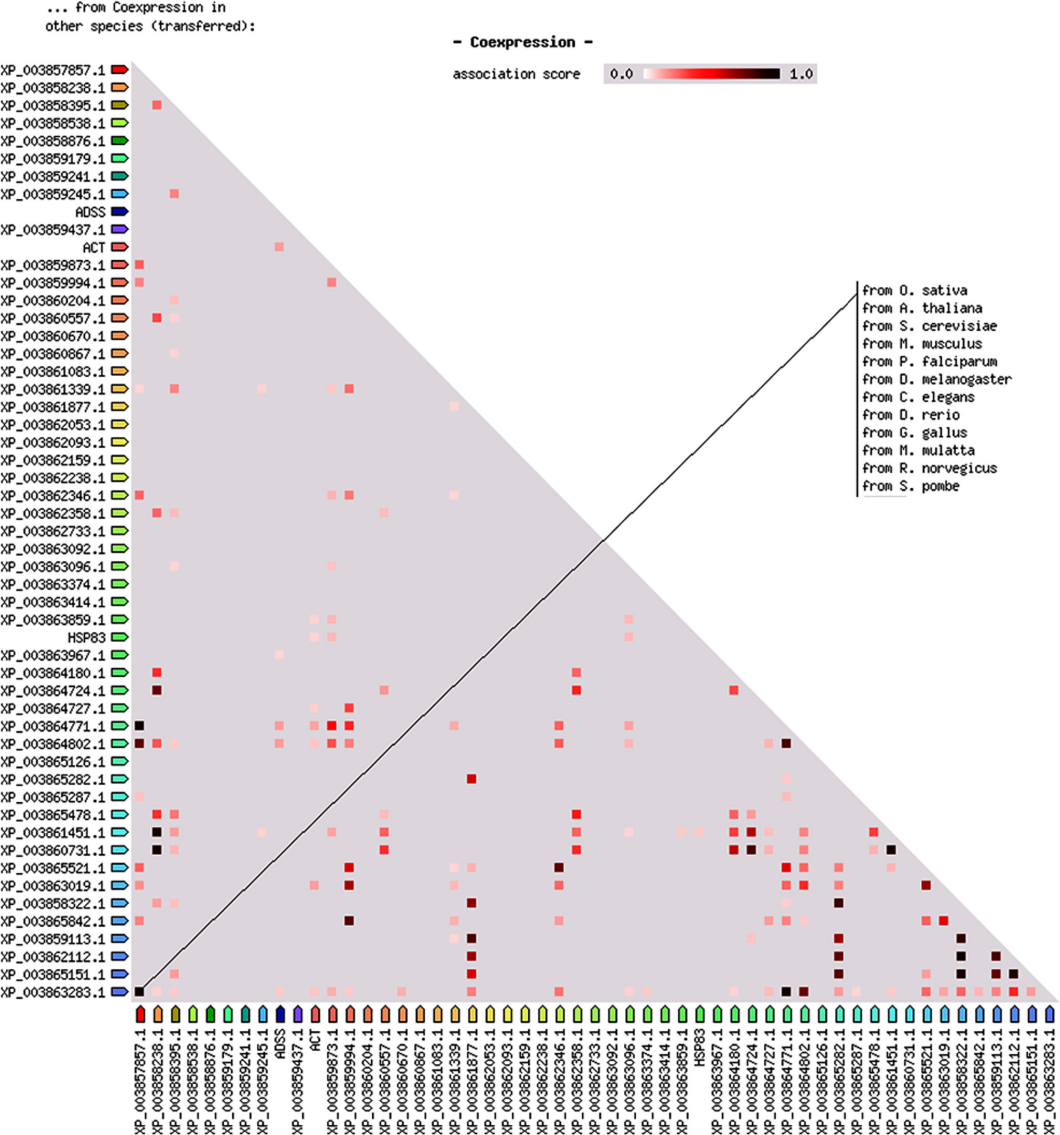
Coexpression analysis of different proteins form the protein-protein interaction network. The coexpression analysis was performed by using STRING based on the data available from different species including *O*. *sativa*, *A*. *thaliana*, *S*. *cerevisiae*, *M*. *musculus*, *P*. *falciparum*, *D*. *melanogaster*, *C*. *elegans*, *D*. *rerio*, *G*. *gallus*, *S*. *pombe*, *M*. *mulatta*, *R*. *norvegicus*.

**Fig 5:**
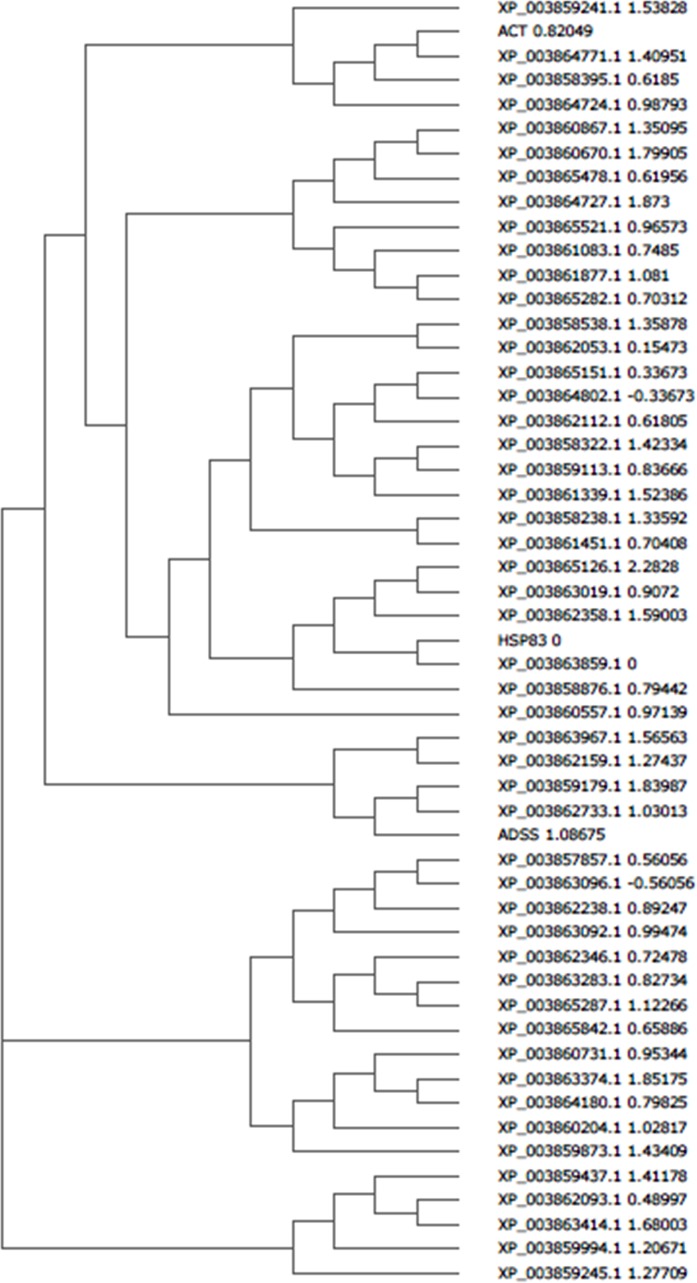
Cladogram showing phylogenetic analysis of different proteins form protein protein interaction network. The phylogenetic correlation between proteins from protein protein interaction network has been analysed by using Clustal W2. The closely related proteins are clustered together whereas distanly related proteins are placed distantly in the cladogram.

Network analysis of the differentially expressed proteins i.e. up regulated proteins (>1.5 fold up regulation) and down regulated proteins (< 0.9 fold down regulation) was performed by using STRING database. The differentially altered proteins were found to be interconnected with each other forming a network shown in Fig 3. List of input proteins used for protein-protein interaction analysis and their predicted functional partners with their accession numbers are mentioned in S1 Table and S2 Table, respectively. The mode of interaction of different proteins is represented by different colours in the interaction network. The directionality of action is represented by the dots present at the end of each connecting line. Fig 4 shows the predicted co-expression of different proteins form protein-protein interaction network and their predicted functional partners to increase the understanding of their correlation. The correlation has been predicted from the data available from different organis like *O*. *sativa*, *A*. *thaliana*, S. *cerevisiae* etc. The phylogenetic relationship between the proteins and their predicted functional partners is shown in Fig 5. The closely related proteins are clustered together whereas distantly related proteins are placed distantly in different clades.

## Discussion

Previously we have analyzed that the spermidine starvation after hypericin treatment induces death of *Leishmania* promastigotes. Further, to understand the potential role of spermidine in processes other than redox metabolism, we have analyzed the complete proteome of the *Leishmania donovani* after hypericin treatment. We have performed label free quantitative proteome analysis to analyze differentially modulated proteins after hypericin treatment. We have observed differential expression of proteins involved in several pathways like protein synthesis, stress and protein folding, metabolic processes, cytoskeleton and cell motility, fatty acids, signalling, transporters and membrane proteins, nucleic acids, hypothetical proteins and protein turnover and modification. The alterations in the proteins involved in a variety of important pathways during spermidine starvation indicate the direct or indirect role of spermidine in these processes.

We have observed increase in expression of Ribosomal protein S7 after hypericin treatment. Ribosomal protein S7 is encoded by str operon. It is reported that ribosomal protein S7 act as repressor of translation. In bacteria, the over expression of ribosomal protein S7 results in translation repression by binding to str operon and causing ultimate reduction in bacterial growth. This repression would result in imbalance between transcription of rRNA and translation of various ribosomal proteins [20]. Spermidine has been found to be important in hypusine modification and hence activity of eIF5A of certain organisms such as *Saccharomyces cerevisiae* [21]. Here, a significant increase in expression of ribosomal protein S7 after spermidine starvation could be a result of altered hypusine modification of eIF5A. Significant decrease in RNA binding protein and translation initiation factor could be a direct result of defective hypusine modification of eIF5A after hypericin treatment. In addition to its role in transcription, RNA binding proteins are also known as regulators of translation [22]. There has been decrease in proteasome alpha 7 subunit and proteasome alpha 1 subunit after hypericin treatment. Inhibition of ubiquitin proteasome system leads to the inhibition of hypusine modification and accumulation of unmodified eIF5A [23]. This further suggests the altered hypusine modification of eIF5A and altered protein synthesis under spermidine starvation after hypericin treatment. A significant increase in elongation factor 1-alpha after hypericin treatment could be a further indication of altered protein synthesis during spermidine starvation.

In our previous study, we have observed that hypericin treatment has increased the formation of reactive oxygen species in *Leishmania* promastigotes [1]. Increase in reactive oxygen species is known to cause DNA damage [24, 25]. Here in this study, we observe altered expression of different stress related proteins like Hsp 90 and Hsp 83–1, stress inducible protein 1 and cyclophilin under spermidine starvation. It is reported that inhibition of Hsp 90 decreases cellular response towards DNA damage due to radiation [26]. Here we have observed increased expression of Hsp 90 after hypericin treatment suggesting response towards stress such as DNA damage induced by ROS generation. There was an increase in expression of stress inducible protein 1 (STI1) which is reported to be a cochaperone of HSP 90 after hypericin treatment. Hypericin treatment has decreased the expression of cyclophilin in *Leishmania* promastigotes. It is reported that mice lacking Ppif gene (forming cyclophilin D) was protected from cell death induced by oxidative stress [27]. These alterations in proteins involved in stress response indicate that the parasite is trying to protect itself from the oxidative stress generated after hypericin treatment. In our previous studies we have observed that hypericin induced death of the parasite is independent of ROS generation [1]. So here these alterations in stress related protein could be a reason of ROS independence of parasite death after hypericin treatment. A very significant increase in histone H2B was also observed. In Saccharomyces cerevisiae, histone H2B was found to be involved in post replication DNA repair [28]. Here, we have observed 5.06 fold increase in the histone H2B expression after hypericin treatment. This increased expression of the histone H2B could be a response of the parasite towards hypericin induced ROS generation.

There were alterations in several other proteins such as those involved in metabolism, fatty acid and nucleic acid related proteins, protein involved in signalling, transporters and membrane proteins and hypothetical proteins. A significant decrease in leishmanolysin protein was observed after hypericin treatment. Leishmanolysin is a glycoprotein present on the surface of *Leishmania* promastigotes. It is involved in infection by establishing parasite’s interaction with the host defence system. It is considered to be a good vaccine candidate [29]. A decrease in leishmanolysin after hypericin treatment is indicative of compromised infectivity of the parasite. ADP ribosylation factor is a GTP binding protein and are known to be involved in vesicular trafficking, internalization of G protein coupled receptors etc [30]. The expression of ADP ribosylation factor was found to be decreased after hypericin treatment which is indicative of altered signalling and membrane trafficking after hypericin treatment. There was significant decrease in C—terminal motor kinesin and flagellar radial spoke protein after hypericin treatment. Kinesin proteins containing motor domain in *Leishmania donovani* is known to be involved in ATP hydrolysis, flagellar movement, microtubule binding, cell division etc [31]. A decrease in expression of C—terminal motor kinesin after hypericin treatment is indicative of altered flagellar movement, ATP hydrolysis and other functions related to this protein. Radial spokes are important structures required for flagellar and cilliary motility of eukaryotic flagella [32]. A decrease in flagellar radial spoke protein after hypericin treatment is again indicative of impaired flagellar movement. Alteration in proteins related to nucleic acid, like universal minicircle sequence binding protein, adenylosuccinate synthetase, dihydrofolate reductase-thymidylate is indicative of altered nucleic acid biosynthesis, metabolism and replication. universal minicircle sequence binding protein (UMSBP) is an important mitochondrial origin protein of trypanosomes which are involved in replication of kinetoplast. In *Trypanosoma brucei*, silencing of UMSBP has resulted in remarkable defects in cell cycle, inhibited replication and minicircle DNA and ultimately resulted arrested the growth [33]. Here, a decrease in UMSBP expression after hypericin treatment could be indicative of altered replication of kinetoplast DNA.

Hypericin treatment has shown significant alteration in different hypothetical proteins present in *Leishmania donovani*. Hypothetical proteins are those proteins whose functions are not yet known. Functional annotation of hypothetical proteins can be done by integrated approach taking in account several factors like conserved domain, sequence identity, phylogenetic relationship, clustering approaches, three dimensional structure etc [34, 35]. However, to give a brief insight, we have found the presence of conserved domains like DEDDy 3'-5' exonuclease domain of *Drosophila egalitarian* (Egl) and similar proteins in hypothetical protein 322501847 (2.62 fold upregulated), HINT (histidine triad nucleotide-binding protein) subgroup in hypothetical protein 322497611 (1.57 fold upregulated), and presence of tetratricopeptide repeat in hypothetical protein 322503307 (2.31 fold up-regulated) (data not shown). Similarly hypothetical protein 322502229 (0.36 fold down-regulation) shows hits with 17-beta-hydroxysteroid dehydrogenases (17beta-HSDs) types -1, -3, and -12, -like, classical (c) SDRs in BLAST analysis (data not shown). Alteration in the expression of these hypothetical proteins is indicative of several altered pathways after hypericin treatment. However, complete functional annotation of hypothetical proteins is required to elucidate the exact pathways altered after hypericin treatment showing the involvement of hypothetical proteins.

Protein-protein interaction analysis of annotated and altered proteins has revealed the interconnection among the proteins altered after hypericin treatment. This indicates that modulation in expression of one protein is tightly correlating the expression of other proteins and vice versa. Mode of interaction of different proteins represents how one protein is involved in the interaction with other i.e. either through binding, reaction or catalysis. Further, co-expression and phylogenetic analysis of the proteins are clear indication of functional relationship of different proteins from protein-protein interaction network and their predicted functional partners. Ultimately, these alterations have led to the disturbed metabolism of the parasite as evident by altered expression of several genes involved in different metabolic pathways and biosynthesis. We have earlier studied redox metabolism of the pathogen and highlighted importance of trypanothione [36–38]. They current work provide importance of another key molecule, spermidine. The quantitative proteome data are available via ProteomeXchange Consortium via the PRIDE [39] with identifier PXD003815.

## Supporting Information

S1 Table List of input protein list for protein interaction analysis with their accession numbers. (DOC)

S2 Table List of predicted functional partners of input protein list used for protein-protein interaction analysis. (DOC)
