# Supplementary material for: Quantitative Proteome Analysis of Leishmania donovani under Spermidine Starvation
Source: PLoS One. 2016 Apr 28;11(4):e0154262. doi: 10.1371/journal.pone.0154262 (PMC4849798; doi:10.1371/journal.pone.0154262)
Supplement: S1 Table — (DOC) [file pone.0154262.s001.doc]

| S1 Table **:** List of input protein list for protein interaction analysis with their accession numbers.  **Accession Number Name of Protein** | | |  |
| --- | --- | --- | --- |
|  | [XP_003857857.1](http://string-db.org/newstring_cgi/display_single_node.pl?taskId=FqW2W6kPFSuz&node=808182&targetmode=proteins) | ribosomal protein S7, putative (200 aa) |
|  | [XP_003858238.1](http://string-db.org/newstring_cgi/display_single_node.pl?taskId=FqW2W6kPFSuz&node=808563&targetmode=proteins) | ATPase alpha subunit; Produces ATP from ADP in the presence of a proton gradient across the membrane (By similarity) (574 aa) |
|  | [XP_003858395.1](http://string-db.org/newstring_cgi/display_single_node.pl?taskId=FqW2W6kPFSuz&node=808720&targetmode=proteins) | dihydrofolate reductase-thymidylate synthase; Bifunctional enzyme. Involved in de novo dTMP biosynthesis. Key enzyme in folate metabolism (By similarity) (520 aa) |
|  | [XP_003858538.1](http://string-db.org/newstring_cgi/display_single_node.pl?taskId=FqW2W6kPFSuz&node=808863&targetmode=proteins) | splicing factor ptsr1-like protein (375 aa) |
|  | [XP_003858876.1](http://string-db.org/newstring_cgi/display_single_node.pl?taskId=FqW2W6kPFSuz&node=809201&targetmode=proteins) | folate/biopterin transporter, putative (691 aa) |
|  | [XP_003859179.1](http://string-db.org/newstring_cgi/display_single_node.pl?taskId=FqW2W6kPFSuz&node=809504&targetmode=proteins) | cytochrome c oxidase subunit IV (343 aa) |
|  | [XP_003859241.1](http://string-db.org/newstring_cgi/display_single_node.pl?taskId=FqW2W6kPFSuz&node=809566&targetmode=proteins) | flagellar radial spoke protein, putative (600 aa) |
|  | [XP_003859245.1](http://string-db.org/newstring_cgi/display_single_node.pl?taskId=FqW2W6kPFSuz&node=809570&targetmode=proteins) | alpha tubulin; Tubulin is the major constituent of microtubules. It binds two moles of GTP, one at an exchangeable site on the beta chain and one at a non-exchangeable site on the alpha chain (By similarity) (451 aa) |
|  | [ADSS](http://string-db.org/newstring_cgi/display_single_node.pl?taskId=FqW2W6kPFSuz&node=809645&targetmode=proteins) | adenylosuccinate synthetase, putative; Plays an important role in the salvage pathway for purine nucleotide biosynthesis. Catalyzes the first committed step in the biosynthesis of AMP from IMP (By similarity) (710 aa) |
|  | [XP_003859437.1](http://string-db.org/newstring_cgi/display_single_node.pl?taskId=FqW2W6kPFSuz&node=809762&targetmode=proteins) | fatty acid elongase, putative (280 aa) |
|  | [ACT](http://string-db.org/newstring_cgi/display_single_node.pl?taskId=FqW2W6kPFSuz&node=810067&targetmode=proteins) | aspartate carbamoyltransferase, putative (327 aa) |
|  | [XP_003859873.1](http://string-db.org/newstring_cgi/display_single_node.pl?taskId=FqW2W6kPFSuz&node=810198&targetmode=proteins) | elongation factor 1-alpha; This protein promotes the GTP-dependent binding of aminoacyl-tRNA to the A-site of ribosomes during protein biosynthesis (By similarity) (449 aa) |
|  | [XP_003859994.1](http://string-db.org/newstring_cgi/display_single_node.pl?taskId=FqW2W6kPFSuz&node=810319&targetmode=proteins) | translation initiation factor, putative (709 aa) |
|  | [XP_003860204.1](http://string-db.org/newstring_cgi/display_single_node.pl?taskId=FqW2W6kPFSuz&node=810529&targetmode=proteins) | C-terminal motor kinesin, putative (841 aa) |
|  | [XP_003860557.1](http://string-db.org/newstring_cgi/display_single_node.pl?taskId=FqW2W6kPFSuz&node=810882&targetmode=proteins) | mitochondrial processing peptidase alpha subunit, putative (467 aa) |
|  | [XP_003860670.1](http://string-db.org/newstring_cgi/display_single_node.pl?taskId=FqW2W6kPFSuz&node=810995&targetmode=proteins) | vesicule-associated membrane protein, putative (257 aa) |
|  | [XP_003860867.1](http://string-db.org/newstring_cgi/display_single_node.pl?taskId=FqW2W6kPFSuz&node=811192&targetmode=proteins) | farnesyl pyrophosphate synthase (362 aa) |
|  | [XP_003861083.1](http://string-db.org/newstring_cgi/display_single_node.pl?taskId=FqW2W6kPFSuz&node=811408&targetmode=proteins) | vacuolar proton translocating ATPase subunit A, putative (775 aa) |
|  | [XP_003861339.1](http://string-db.org/newstring_cgi/display_single_node.pl?taskId=FqW2W6kPFSuz&node=811664&targetmode=proteins) | poly(A)-binding protein, putative (544 aa) |
|  | [XP_003861877.1](http://string-db.org/newstring_cgi/display_single_node.pl?taskId=FqW2W6kPFSuz&node=812202&targetmode=proteins) | proteasome alpha 7 subunit, putative (238 aa) |
|  | [XP_003862053.1](http://string-db.org/newstring_cgi/display_single_node.pl?taskId=FqW2W6kPFSuz&node=812378&targetmode=proteins) | RNA-binding protein, putative (403 aa) |
|  | [XP_003862093.1](http://string-db.org/newstring_cgi/display_single_node.pl?taskId=FqW2W6kPFSuz&node=812418&targetmode=proteins) | protein kinase, putative (560 aa) |
|  | [XP_003862159.1](http://string-db.org/newstring_cgi/display_single_node.pl?taskId=FqW2W6kPFSuz&node=812484&targetmode=proteins) | major surface protease gp63, putative (566 aa) |
|  | [XP_003862238.1](http://string-db.org/newstring_cgi/display_single_node.pl?taskId=FqW2W6kPFSuz&node=812563&targetmode=proteins) | phenylalanine-4-hydroxylase, putative (452 aa) |
|  | [XP_003862346.1](http://string-db.org/newstring_cgi/display_single_node.pl?taskId=FqW2W6kPFSuz&node=812671&targetmode=proteins) | eukaryotic translation initiation factor, putative; Component of the eukaryotic translation initiation factor 3 (eIF-3) complex, which is involved in protein synthesis and, together with other initiation factors, stimulates binding of mRNA and methionyl-tRNAi to the 40S ribosome (By similarity) (405 aa) |
|  | [XP_003862358.1](http://string-db.org/newstring_cgi/display_single_node.pl?taskId=FqW2W6kPFSuz&node=812683&targetmode=proteins) | 2-oxoglutarate dehydrogenase, E2 component, dihydrolipoamide succinyltransferase, putative (389 aa) |
|  | [XP_003862733.1](http://string-db.org/newstring_cgi/display_single_node.pl?taskId=FqW2W6kPFSuz&node=813058&targetmode=proteins) | 2-hydroxy-3-oxopropionate reductase, putative (299 aa) |
|  | [XP_003863092.1](http://string-db.org/newstring_cgi/display_single_node.pl?taskId=FqW2W6kPFSuz&node=813417&targetmode=proteins) | 5-methyltetrahydropteroyltriglutamate-homocysteine S-methyltransferase, putative (770 aa) |
|  | [XP_003863096.1](http://string-db.org/newstring_cgi/display_single_node.pl?taskId=FqW2W6kPFSuz&node=813421&targetmode=proteins) | cyclophilin, putative; PPIases accelerate the folding of proteins (By similarity) (229 aa) |
|  | [XP_003863374.1](http://string-db.org/newstring_cgi/display_single_node.pl?taskId=FqW2W6kPFSuz&node=813699&targetmode=proteins) | ADP-ribosylation factor, putative (178 aa) |
|  | [XP_003863414.1](http://string-db.org/newstring_cgi/display_single_node.pl?taskId=FqW2W6kPFSuz&node=813739&targetmode=proteins) | protein kinase, putative (429 aa) |
|  | [XP_003863859.1](http://string-db.org/newstring_cgi/display_single_node.pl?taskId=FqW2W6kPFSuz&node=814184&targetmode=proteins) | heat shock protein 83-1 (700 aa) |
|  | [HSP83](http://string-db.org/newstring_cgi/display_single_node.pl?taskId=FqW2W6kPFSuz&node=814185&targetmode=proteins) | heat shock protein 83-1; Molecular chaperone that promotes the maturation, structural maintenance and proper regulation of specific target proteins involved for instance in cell cycle control and signal transduction. Undergoes a functional cycle that is linked to its ATPase activity. This cycle probably induces conformational changes in the client proteins, thereby causing their activation. Interacts dynamically with various co-chaperones that modulate its substrate recognition, ATPase cycle and chaperone function (By similarity) (700 aa) |
|  | [XP_003863967.1](http://string-db.org/newstring_cgi/display_single_node.pl?taskId=FqW2W6kPFSuz&node=814292&targetmode=proteins) | cysteine conjugate beta-lyase, aminotransferase-like protein (414 aa) |
|  | [XP_003864180.1](http://string-db.org/newstring_cgi/display_single_node.pl?taskId=FqW2W6kPFSuz&node=814505&targetmode=proteins) | malate dehydrogenase (317 aa) |
|  | [XP_003864724.1](http://string-db.org/newstring_cgi/display_single_node.pl?taskId=FqW2W6kPFSuz&node=815049&targetmode=proteins) | mitochondrial processing peptidase, beta subunit, putative (490 aa) |
|  | [XP_003864727.1](http://string-db.org/newstring_cgi/display_single_node.pl?taskId=FqW2W6kPFSuz&node=815052&targetmode=proteins) | threonyl-tRNA synthetase, putative (788 aa) |
|  | [XP_003864771.1](http://string-db.org/newstring_cgi/display_single_node.pl?taskId=FqW2W6kPFSuz&node=815096&targetmode=proteins) | ribosomal protein L15, putative (204 aa) |
|  | [XP_003864802.1](http://string-db.org/newstring_cgi/display_single_node.pl?taskId=FqW2W6kPFSuz&node=815127&targetmode=proteins) | 60S ribosomal protein L12, putative (164 aa) |
|  | [XP_003865126.1](http://string-db.org/newstring_cgi/display_single_node.pl?taskId=FqW2W6kPFSuz&node=815451&targetmode=proteins) | stress-inducible protein STI1 homolog (255 aa) |
|  | [XP_003865282.1](http://string-db.org/newstring_cgi/display_single_node.pl?taskId=FqW2W6kPFSuz&node=815607&targetmode=proteins) | proteasome alpha 1 subunit, putative; The proteasome is a multicatalytic proteinase complex which is characterized by its ability to cleave peptides with Arg, Phe, Tyr, Leu, and Glu adjacent to the leaving group at neutral or slightly basic pH. The proteasome has an ATP-dependent proteolytic activity (By similarity) (264 aa) |
