# Supplementary material for: Quantitative Proteome Analysis of Leishmania donovani under Spermidine Starvation
Source: PLoS One. 2016 Apr 28;11(4):e0154262. doi: 10.1371/journal.pone.0154262 (PMC4849798; doi:10.1371/journal.pone.0154262)
Supplement: S2 Table — (DOC) [file pone.0154262.s002.doc]

| S2 Table **:** List of predicted functional partners of input protein list used for protein-protein interaction analysis.  **Accession Number Name of Protein** | | |  |  |
| --- | --- | --- | --- | --- |
|  | [XP_003861451.1](http://string-db.org/newstring_cgi/display_single_node.pl?taskId=FqW2W6kPFSuz&node=811776&targetmode=proteins) | hypothetical protein; (525 aa) |  | |
|  | [XP_003860731.1](http://string-db.org/newstring_cgi/display_single_node.pl?taskId=FqW2W6kPFSuz&node=811056&targetmode=proteins) | ATP synthase F1 subunit gamma protein, putative (303 aa) |  | |
|  | [XP_003865521.1](http://string-db.org/newstring_cgi/display_single_node.pl?taskId=FqW2W6kPFSuz&node=815846&targetmode=proteins) | eukaryotic translation initiation factor 3 subunit, putative (356 aa) |  | |
|  | [XP_003863019.1](http://string-db.org/newstring_cgi/display_single_node.pl?taskId=FqW2W6kPFSuz&node=813344&targetmode=proteins) | eukaryotic translation initiation factor 3 subunit 7-like protein (531 aa) |  | |
|  | [XP_003858322.1](http://string-db.org/newstring_cgi/display_single_node.pl?taskId=FqW2W6kPFSuz&node=808647&targetmode=proteins) | proteasome beta 6 subunit, putative (247 aa) |  | |
|  | [XP_003865842.1](http://string-db.org/newstring_cgi/display_single_node.pl?taskId=FqW2W6kPFSuz&node=816167&targetmode=proteins) | eukaryotic translation initiation factor 3 subunit 8, putative (731 aa) |  | |
|  | [XP_003859113.1](http://string-db.org/newstring_cgi/display_single_node.pl?taskId=FqW2W6kPFSuz&node=809438&targetmode=proteins) | hypothetical protein; (283 aa) |  | |
|  | [XP_003862112.1](http://string-db.org/newstring_cgi/display_single_node.pl?taskId=FqW2W6kPFSuz&node=812437&targetmode=proteins) | proteasome beta 3 subunit, putative (205 aa) |  | |
|  | [XP_003865151.1](http://string-db.org/newstring_cgi/display_single_node.pl?taskId=FqW2W6kPFSuz&node=815476&targetmode=proteins) | proteasome beta 2 subunit, putative (206 aa) |  | |
|  | [XP_003863283.1](http://string-db.org/newstring_cgi/display_single_node.pl?taskId=FqW2W6kPFSuz&node=813608&targetmode=proteins) | ubiquitin-fusion protein (128 aa) |  | |
